# Green construction and release mechanism of lignin-based double-layer coated urea

**DOI:** 10.1186/s13068-023-02355-7

**Published:** 2023-06-08

**Authors:** Xiaojuan Chen, Huchen Yang, Lidan Zhang, Zhongli Li, Yunna Xue, Rongfeng Wang, Xiaolin Fan, Shaolong Sun

**Affiliations:** College of Natural Resources and Environment, South China Agricultural University, Guangzhou, 510642 Guangdong China

**Keywords:** Green construction, Double layer, Lignin-based polyurethane, Coated urea, Release mechanism

## Abstract

**Background:**

Lignin played an important role in the establishment of coated fertilizers coating material as a substitute for petrochemical raw materials. However, so far, the lignin-based coated fertilizers was limited in only the poor slow-release performance. To achieve good slow-release performance of lignin-based coated fertilizers, hydrophilic of lignin need to be resolved to establish an green and better controllable lignin-based coated fertilizers.

**Results:**

In the study, a novel green double layer coating with lignin-based polyurethane (LPU) as the inner coating and epoxy resin (EP) as the outer coating was effectively constructed for coated urea. Fourier transform infrared spectra confirmed that lignin and polycaprolactone diol successfully reacted with Hexamethylene diisocyanate. The loss weight and water contact angle (WCA, 75.6–63.6°) of the LPUs decreased with the increased lignin content. The average particle hardness of the lignin-based double-layer coated urea (LDCU) first increased from 58.1 N (lignin of 30%) to 67.0 N (lignin of 60%), but then decreased to 62.3 N (lignin of 70%)*.* The release longevity of the coated urea was closely related to the preparation parameters of the coating material. The optimal cumulative nutrient release rate (79.4%) of LDCU was obtained (lignin of 50%, –CNO/–OH molar ratios of 1.15, EP of 35%, and coating ratio of 5%). The aggregates of hydrone on the LDCU caused the dissolution and swelling of nutrients, and then the diffusion of nutrients through the concentration gradient.

**Conclusions:**

A though the nutrient release of the LDCUs was affected by many factors, the successful development of the LDCUs will help improve the rapid development of the coated fertilizer industry.

## Background

Fertilizer is an indispensable part of agricultural practices, which help to increase crop yields and sustainable food production [1, 2]. However, due to the large amount of conventional chemical fertilizers and low nutrient utilization, it not only causes a large amount of nutrient loss, greenhouse gas emissions (CO_2_, CH_4_, N_2_O) and waste of resources, but also leads to serious environmental pollution and threat to human health, which hinders the sustainable development of agriculture [3–5]. The development of coated controlled release fertilizer is considered to be a method that can significantly improve the efficiency of nutrient and minimize the environmental hazards [6, 7]. It is composed of core fertilizer and coated layer. Coating layers are divided into inorganic materials (such as sulfur and other minerals) and organic polymer materials (such as thermoplastics and resins). The latter has been used for a long time because of its excellent performance [8]. However, most organic polymer materials come from petroleum, which has problems, such as expensive, non-renewable, and non-degradable [9]. Therefore, it is particularly important to develop cheap, renewable and degradable coating layers.

In the past few decades, researchers have focused on developing new polymers to produce coated controlled-release fertilizers using green, renewable and degradable resources as raw materials, such as starch [10], cellulose [11], chitosan [12], and vegetable oil [13–15]. However, these degradable resources as coating materials for coated fertilizers have certain disadvantages. For example, vegetable oils and starches compete with humans for food, while cellulose and chitosan are more expensive, which greatly limits their practical applications. As an important component of plant dry weight, lignin has the advantages of green, abundant and degradable [16–18]. The pulp and paper industry is known as the main source of technical lignin (TL). Currently, 50–70 million tons of TL was produced worldwide every year [19–22]. It is estimated that the value will increase to 225 million tons per year by 2030 as the annual production of TL (a by-product of biofuel production) increases. However, due to the low purity and complex structure of TL, its application in coating materials of fertilizer is limited. In recent years, TL was directly mixed with plasticizers or hydrophobic agents (paraffin, ethyl cellulose, rosin, starch, polylactic acid) through physical compounding methods, and then coated on fertilizers [23–25]. However, the sustained release time of the prepared coated fertilizer did not meet the release standard due to the poor coating effect through these methods. For example, four different sources of TL (two lignosulfonates, one lignin sulfate, one linolein lignin) and hydrophobic compounds (alkenyl succinic anhydride, glycerol, lupranol, acronal, styronal, sorbitol, PEG400, PEG2000, PEG6000, alkenyl succinic anhydride, 2-2-phosphinicobis-butanedioic acid) were mixed as coating materials to prepare coated urea [26]. The results showed that the coating material prepared by sulfur-free soda lignin had the best viscosity and film-forming properties under alkaline conditions. Nevertheless, the prepared coated fertilizer appeared poor slow-release performance, that was, approximately 80% release of urea in 25 min through the hydrostatic test. In addition, continuous chemical modification (methylolation, acetylation, esterification, etc.) and different degrees of physicochemical treatment of lignin were applied to obtain lignin-based coating materials, which were then coated on the surface of the core fertilizer. For example, lignin extracted from kraft paper and sulfite black liquor in the pulp and paper industry was used as the raw material, which was then acetylated and wrapped in urea [27]. The results showed that the maximum time for the prepared coated urea to release 80% of nitrogen in water and soil was about 50 h and 20 days, respectively. Li et al. [28] evaluated the effects of esterification and acetylation of industrial sulfate lignin and polylactic acid (PLA dichloro methane-dioxane as solvent) coated on urea. The functionalized lignin delayed the release of urea in water. However, the large-scale application of lignin-based coated materials was limited due to the complexity of the process and the high cost during most lignin modification processes and the poor slow-release performance in lignin-based coated fertilizers. At the same time, the modification processes usually added toxic organic solvents, which caused environmental pollution and endangered human health [29]. Furthermore, bio-based polyurethane materials are recognized as coating materials of fertilizer. Due to the abundant hydroxyl groups in the lignin structure, it can partially replace petroleum-based raw materials and react with isocyanates to generate lignin-based polyurethane (LPU) for preparing controlled release urea (CRU) coating materials. However, the LPU prepared by adding a large amount of unmodified lignin was both brittle and hard due to the inherent rigidity of lignin itself and the low molecular mobility of diisocyanates [30]. Therefore, it is very necessary to develop a low-cost, green (without any toxic organic solvents) and better controllable coating materials by adding appropriate lignin as layer material to improve nutrient use efficiency. In addition, epoxy resin (EP) can be used to prepare coating materials to slow down the release rate of fertilizer nutrients due to its excellent properties, such as high bonding strength and good water resistance [31]. Currently, there are few reports about the sustained-release time of single-layer non-modified lignin-based coated urea that meets the sustained-release standard. Therefore, the unmodified lignin-based material by adding a hydrophobic polymer can improve the hydrophobicity of the coating material.

In the current research, a novel green double layer coating technology with LPU as the inner coating and EP as the outer coating was effectively constructed for coated urea. The impacts of lignin content, –CNO/–OH molar ratio, amount of EP, and coating ratio on the nutrient release of lignin-based double-layer coated urea (LDCU) were systematically investigated using a central composite design of response surface approach. Physicochemical properties of the coating layer and LDCU were also evaluated. Based on the above results, release mechanism of nitrogen in the LDCU was revealed. The successful preparation of the LDCU will provide theoretical basis and technical support for the efficient development of coated fertilizer, which is of great significance for promoting the green and sustainable development of the fertilizer industry and agricultural production.

## Results and discussion

### The relationship between parameter of coating material and nutrient release characteristics of the LDCUs

**Fig. 1:**
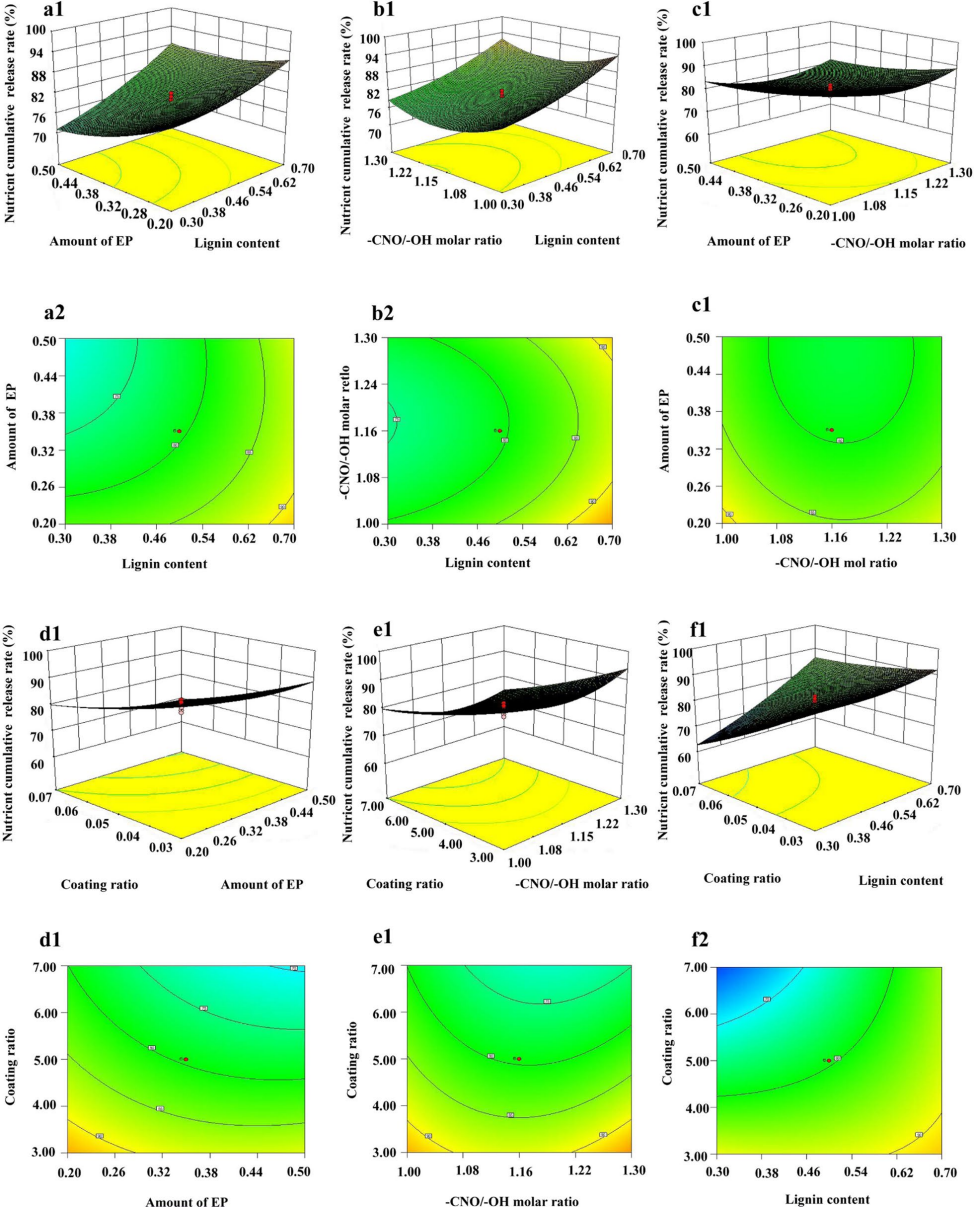
Simulated 3D modeling of AC (**a1**), AB (**b1**), BC (**c1**), CD (**d1**), BD (**e1**), AD (**f1**), and interaction contour maps of factors of AC (**a2**), AB (**b2**), BC (**c2**), CD (**d2**), BD (**e2**), AD (**f2**). A: lignin content; B: -CNO/-OH molar ratio; C: amount of EP; and D: coating ratio

The effects of lignin content, –CNO/–OH molar ratio, amount of EP, and coating ratio on the design array of variable *Y* for nutrient release characteristics of the LDCUs were evaluated (Fig. 1). The model was selected according to the significance of the response value. The process order of the nitrogen cumulative release rate for 35 days was quadratic. The resulting models relating lignin content (*A*), –CNO/–OH molar ratio (*B*), amount of EP (*C*), and coating ratio (*D*) were determined as follows:

$$ {Y} = 79.5 + {6.62A} - {0.96B} - {3.71C} - {8.04D} - {0.062AB} + {2.19AC} + {5.19AD} + {0.19BC} - {1.56BD} - {1.56CD} + {{1.93A}^{2}} + {{4.80B}^{2}} + {{2.18C}^{2}} + {{1.18D}^{2}} $$

Here, *Y* was the nitrogen cumulative release rate for 35 days. *A* (lignin content), *B* (–CNO/–OH molar ratio), *C* (amount of EP), and *D* (coating ratio) were the independent variables. The correlation coefficients *R*^2^ and adjusted *R*^2^ were calculated. The latter coefficient reflected an adjustment for the number of model parameters relative to the number of points. The variance analysis of the model equations showed that the polynomial model used in this experiment was extremely significant, as the *P* value for *Y* was < 0.0001 (*P* < 0.05). The correction coefficient *R*^2^ of the model for *Y* was 94.09%, the lack of fit *Y* was 0.0974 (*P* > 0.05). The data indicated that the N release rate (35 days) of the LDCUs was accurately predicted using the three-dimensional (3D) fitting equation. Central composite design of response surface methodology experiments revealed the following sequence of the nitrogen cumulative release rate for 35 days: coating ratio > content of lignin > amount of EP > –CNO/–OH molar ratio. All of the statistical analyses results showed that AC and AD items interacted with each other on the long-term cumulative release rate and release period.

The fitting relationship between the variable and the response value (*a*1–*f*1) as well as contour lines (*a*2–*f*2) are shown in Fig. 1. The denser the contour, the more the response value was affected by the factor 3D. By response surface analysis, the industry standard for controlled release fertilizer and the cost of the film material. The optimal cumulative nutrient release rate (79.4%) of LDCU was obtained (lignin of 50%, –CNO/–OH molar ratios of 1.15, EP of 35%, and coating ratio of 5%). This result may be related to less material gaps or holes in the coating material. The coated fertilizer with few holes showed a longest longevity [34]. Based on the optimal conditions, the physical and chemical properties of the coating material and the LDCUs were systematically evaluated.

**Fig. 2:**
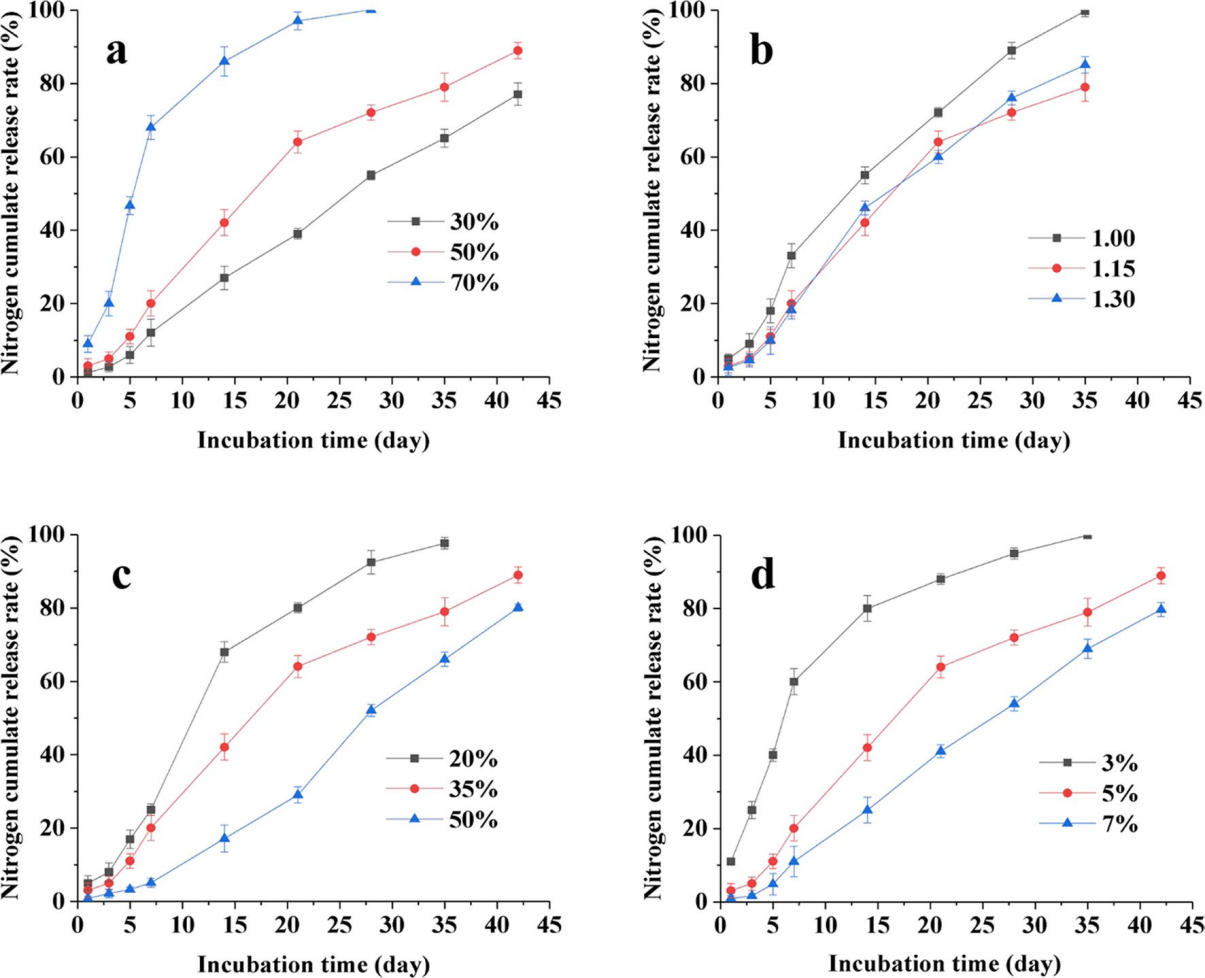
Nitrogen cumulative release rate of the DLCU obtained under the different conditions (**a**: lignin content; **b**: -CNO/-OH molar ratio; **c**: amount of EP; **d**: coating ratio)

The nitrogen release characteristics of the LDCUs were significantly affected by the different parameters (a: lignin content; b: –CNO/–OH molar ratio; c: amount of EP; d: coating ratio) (Fig. 2). The lignin content was directly negative correlated to the longevity of the LDCUs based on nitrogen cumulative release rate (Fig. 2a). The LDCUs prepared with 30% lignin content showed that the nitrogen cumulative release rate of the LDCUs reached more than 80% during the 45 days of incubation. The reason for this was mainly that the change of the type and content of hydroxyl due to the different lignin content. Meanwhile, the longevity of the LDCUs was also closely related to the –CNO/–OH molar ratio (Fig. 2b). The longevity of the LDCUs first increased from 24 days (–CNO/–OH molar ratios of 1.00) to 36 days (–CNO/–OH molar ratios of 1.15), but then decreased to 32 days in the experiment performed at –CNO/–OH molar ratios of 1.30, suggesting that the weakening of mechanical properties of coating material occurred when the –CNO/–OH molar ratios was higher than 1.15. It was noted that a strong positive correlation was obtained between the longevity of the LDCUs (20–42 days) and amount of EP (20–50%) (Fig. 2c). A similar phenomenon was also found between the longevity of the LDCUs and coating ratio (Fig. 2d).

### Physicochemical properties of coating materials

**Fig. 3:**
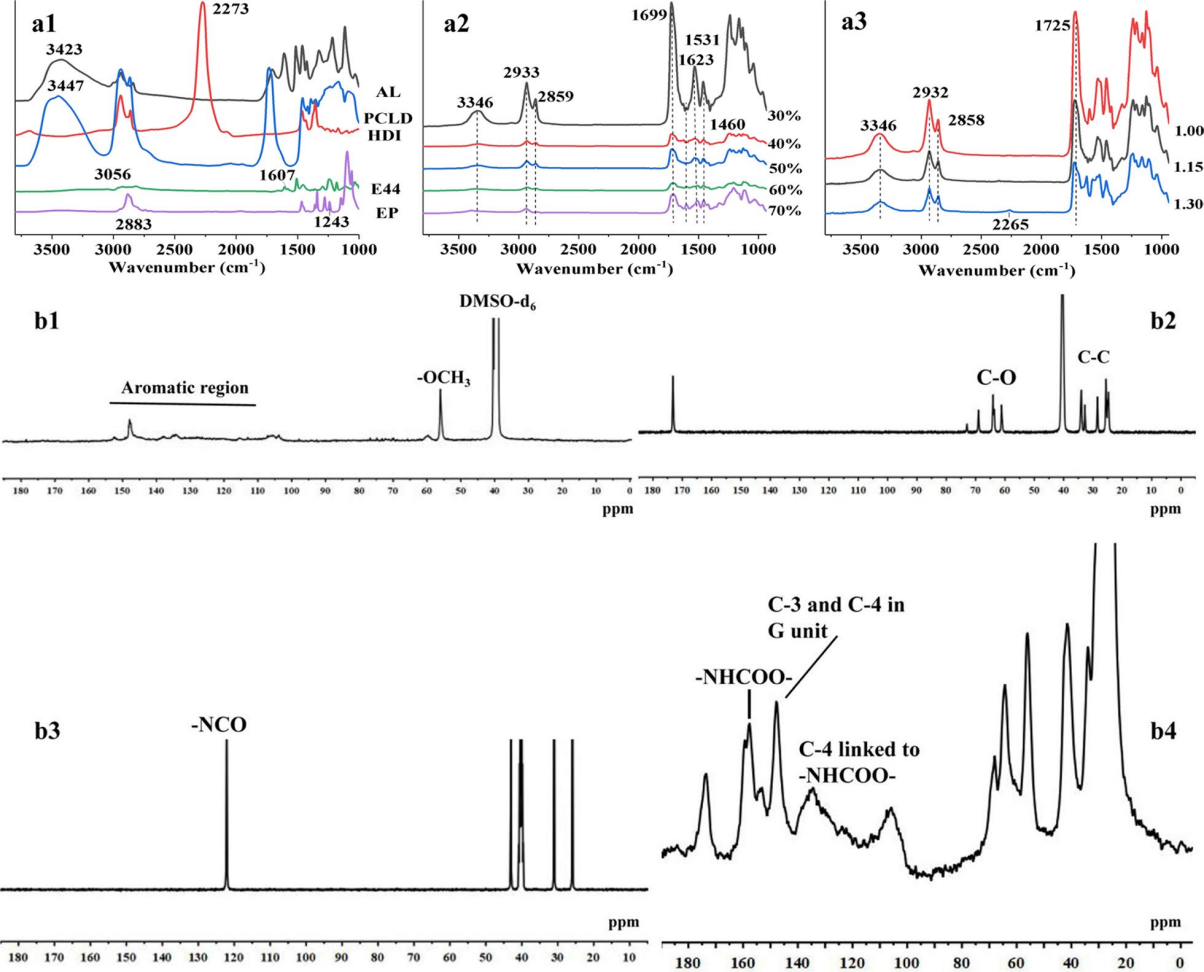
FT-IR analysis of the AL, PCLD, HDI, E44, EP (**a1**), and the LPUs obtained under the different conditions (**a2**: lignin content; **a3**: -CNO/-OH molar ratio). 13C NMR spectra of AL (**b1**), PCLD (**b2**), HDI (**b3**) and the LPU (**b4**)

Figure 3 exhibits the chemical structural changes of the AL, PCLD, HDI, E-44, EP, and LPUs by FT-IR spectra during the syntheses of LPUs and EP (a1–3). Figure 3a1 displays the FT-IR spectra of the AL, PCLD, HDI, E44, and EP. The intense bands at 1607, 1512, and 1425 cm^−1^ correspond to aromatic skeletal vibrations of AL [35]. The strong intensity of the carbonyl absorption band at 1731 cm^−1^ typified the carbonyl absorption of PCLD polyester. The carbonyl groups of lignin usually have a broad absorption band at 1700–1680 cm^−1^. However, the hydroxyl-stretching mode of lignin was weaker than that of PCLD due to the low concentration of lignin hydroxyl chain-end groups. The characteristic band of HDI (–CNO) was observed at 2273 cm^−1^. The bands at 3056 and 1607 cm^−1^ originate from the C–C stretching vibration of E44 (the benzene ring). The two characteristic absorption bands of the EP at 2883 and 1243 cm^−1^ corresponded to the stretching vibrations of the C–H groups and C–O bonds, respectively. The intensity of bands at 3346 cm^−1^ (–OH stretching vibration) and 2859–2933 cm^−1^ (C–H vibration) reduced with increasing lignin content in LPU (Fig. 3a2). The fact was possibly related to the vibration intensity of –OH in AL lower than that of PCLD (Fig. 3a1). It was found that the bands at 2260–2280 cm^−1^ (–CNO groups in HDI) were disappeared in the all spectra of LPU, implying that HDI was totally consumed after the reaction with polyols [36]. In addition, the intensity of band at 3400 cm^−1^ (–OH stretching vibration) reduced with increasing –CNO/–OH molar ratios, indicating that the –OH of AL and PCL reacted strongly with –NCO (Fig. 3a3). A small band appeared at 2265 cm^−1^ under a high –CNO/–OH molar ratio condition, indicating that the incomplete reaction of –NCO (HDI) and –OH (AL and PCLD). This may be related to the complex hydroxyl type of lignin. It is well-known that not all –OH groups from lignin molecules are easily accessible for the reaction with other groups. In addition, phenolic, guaiacyl and syringyl –OH were less reactive towards –NCO group compared to the aliphatic –OH [37, 38]. In short, the LPUs were successfully prepared by reactions of the HDI and polyols (AL and PCLD) under the given conditions.

To further confirm the formation of LPU coating, ^13^C NMR was employed to detect the change of chemical shift of C atoms in raw coating materials (AL, PCLD, and HDI) and formed LPU coating (Fig. 3b1–4). For AL (Fig. 3b1), the characteristic peaks at 103–107 ppm, 134–137 ppm and 147–148 ppm correspond to the C–C and C–O (the aromatic region) of benzene rings, respectively. Meantime, the peaks at 56 ppm belong to the –OCH_3_ of AL. For PCLD (Fig. 3b2), the peaks at chemical shifts of 30–40 ppm and 60–72 ppm are assigned to the C–C and C–O (carbons adjacent to the ester oxygens) bonds, respectively. For HDI (Fig. 3b3), the signals at 122.11 ppm was associated with hydrocarbons of the isocyanate structure (–CNO). For LPU coating (Fig. 3b4), a few characteristic peaks attributed to AL, PCLD, and HDI still can be observed. Compared with the ^13^C NMR spectra of HDI, the disappeared peak at 122.11 ppm which can be attributed to the carbonyl carbons of isocyanate groups revealed that the polymerization reaction had proceeded thoroughly. Furthermore, the appearance of new signals 56–68 ppm corresponded to hydrocarbons of the isocyanate structure, whereas the reaction between the isocyanate and the hydroxyl groups from AL was evidenced by the appearance of new signals corresponding to urethane (–NHCOO–) linkages at 157.56 and 159.54 ppm (secondary and primary urethane linkages, respectively). The new signal appearing at 129.1 ppm was attributed to C-4 from G unit connected to –NHCOO–. These facts further indicated the successful fabrication of LPU coating.

**Fig. 4:**
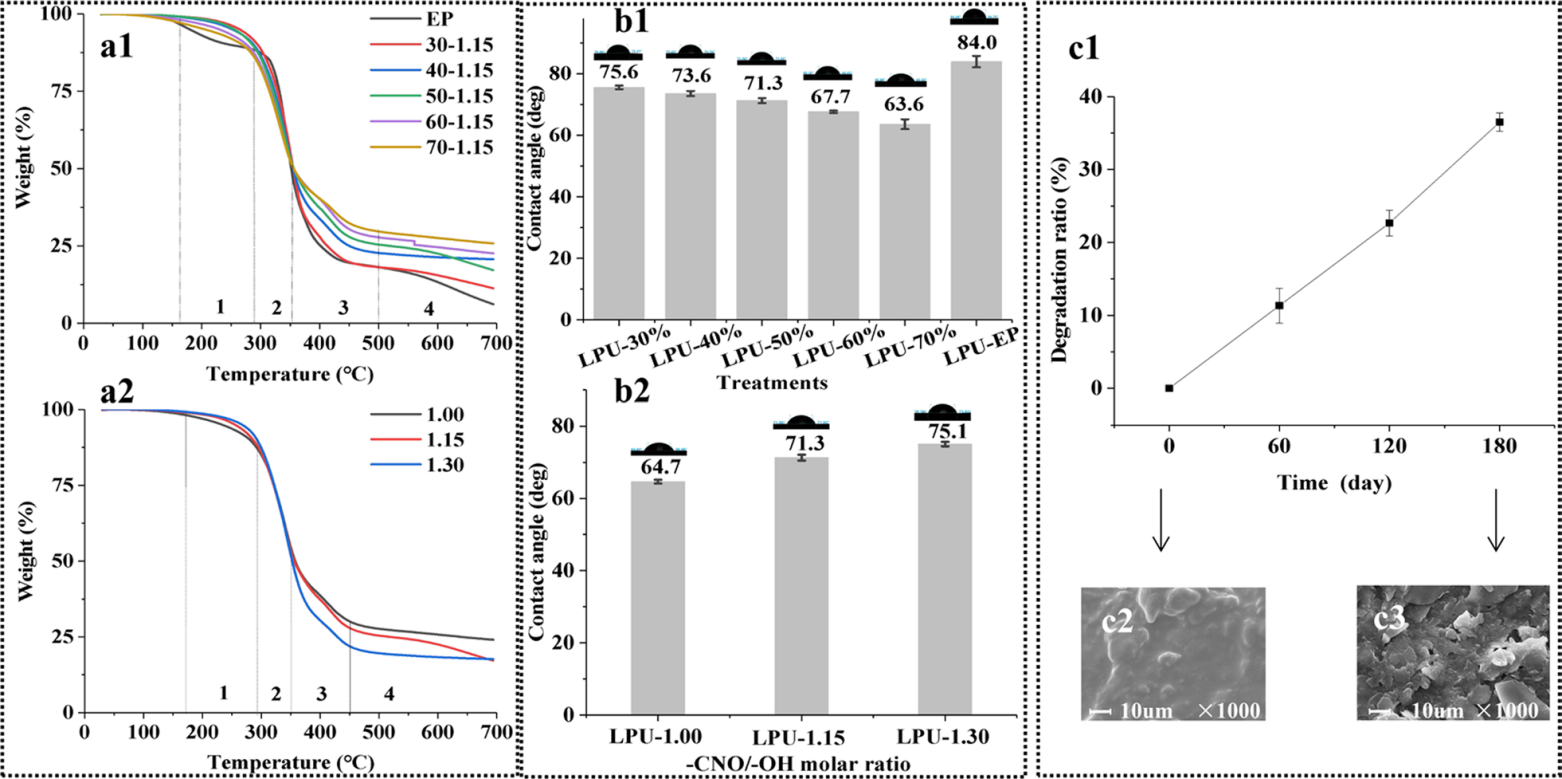
TGA weight loss curves of the LPUs with different lignin content and EP (**a1**), and different -CNO/-OH molar ratio (**a2**). WCA of the LPUs with different lignin content and LPU-EP (**b1**), and different -CNO/-OH molar ratio (**b2**). The degradation ratio of the coating layer in soil (**c1**). SEM images of the 0 (**c2**) and after 180 days (**c3**) coating layer of the LDCU in the soil

To investigate the potential relationship between structural features and thermal behaviors, the TGA of the LPUs were comparatively investigated (Fig. 4a1–2). The thermal degradation processes of the LPUs were mainly divided into four temperature stages, which were 137–285 °C, 285–351 °C, 351–450 °C and higher than 450 °C (Fig. 4a1–2). As can be seen, EP had worse thermal stability than the LPUs. During the first stage of thermal degradation, the weight of LPUs was reduced by 15%, which was mainly caused by the hydroxyl dehydration reaction [39]. The second stage of thermal degradation was the fastest weight loss (about 36%) process due to the degradation of the unstable carbamate groups in LPUs [40]. At this stage, the content of lignin had no obvious effect on the weight loss of LPUs (Fig. 4a1). During the third stage, the weight of the LPUs reduced (33.56–19.37%) with the increasing lignin content (30–70%). The weight of all LPUs was mainly closely related to the decomposition of PCLD ester bonds in LPU and the rupture of carbon–carbon bonds between lignin structural units [41]. This reduced trend was attributed to an increased benzene ring and carbon of lignin in the LPUs [42, 43]. For the last stage, the weight loss of the LPUs was mainly related to the further thermal oxidation of the LPUs. This stage and the third stage showed the same change trend, that was, the weight loss of the LPUs reduced with the increasing lignin content. This similar behavior was founded in previous literature. Llovera et al. [44] synthesized lignin-based polyurethane film using 4, 4-diphenylmethane diisocyanate and polyethylene glycol as raw materials, and analyzed the thermal stability of lignin-based polyurethane film by thermogravimetric analysis. It was found that the weight loss of the LPUs was reduced with the increasing lignin content (0–70%). It was worth noting that the effects of –CNO/–OH molar ratios and lignin content on the weight loss of the LPUs appeared the same trend in the first and second stages during thermal degradation processes (Fig. 4a2). In the third and fourth stages, the weight loss of the LPUs decreased with reducing –CNO/–OH molar ratios. This was similar to the pervious results [45]. In other words, decreasing –CNO/–OH molar ratios caused continued improvement of the thermal stability of the LPUs.

The WCAs were performed to determine the wettability properties of surfaces of the LPUs (Fig. 4b1–2). The wettability of the LPUs was determined by the hydroxyl groups on the surface under the same –CNO/–OH molar ratio condition. The WCAs of the LPUs gradually decreased from 75.6° to 63.6° due to the presence of a large number of carboxyl and hydroxyl groups with the increase of the lignin content (30–70%) (Fig. 4b1). In addition, the value of –CNO/–OH also significantly affected the WCAs of the LPUs (Fig. 4b2). As can be seen, the WCAs of the LPUs (64.7–75.1°) increased with the raise of –CNO/–OH molar ratio, indicating that the wettability of the LPUs was weakened [46]. Zhang et al. reported that the –CNO/–OH molar ratio was positively correlated to the WCAs [47]. The increment of the WCAs was mainly attributed to the efficient reaction of the –OH with –CNO during the preparation of LPUs. Meanwhile, the reaction increased the content of hard segments in the LPUs and then limited the wettability of LPUs [48]. It was noted that the EP-LPU had a higher WCA (84°) than the LPUs (63.6–75.6°). A possible reason was that the EP resulted in reduced the penetration of water molecules due to block the pores of LPU [14], which could prevent water from entering the coating shells and thus slow down *N* release.

To assess the biodegradability of the coating layer of LDCU, the degradation ratio of the coating layer in soil is presented in Fig. 4c1. The degradation ratio of the coating layer increased with the soil burial time prolonged. As expected, the degradation ratio of the coating layer reached a maximum value (36.5%) after 180 days, suggesting that the prepared coating layer was an environmentally friendly and degradable material. Furthermore, this was similar to the results of the biodegradability of lignin–poly(ε-caprolactone)-based PU [41]. The SEM images of the 0 (*c*2) and degraded after 180 days (*c*3) coating layer of the LDCU in the soil are also presented in Fig. 3. Compared with the initial dense structure, the dense structure of the coating layer was destroyed and exhibited fragmented structures of different sizes after 180 days of degradation. In general, the soil environment contains abundant microorganisms (bacteria and fungi), which promote the hydrolysis of the coating layer in the LDCU [49]. In addition, this may also be related to the decomposition of lignin macromolecular structure, biodegradable LDCU and epoxy resin in the soil by microorganisms and enzymes [50]. In summary, the coating layer of the LDCU appeared excellent biodegradability in the soil, suggesting that lignin could be widely applied in the layer construction of coated fertilizer.

### Surface properties, physical properties and swelling performance of the LDCUs

**Fig. 5:**
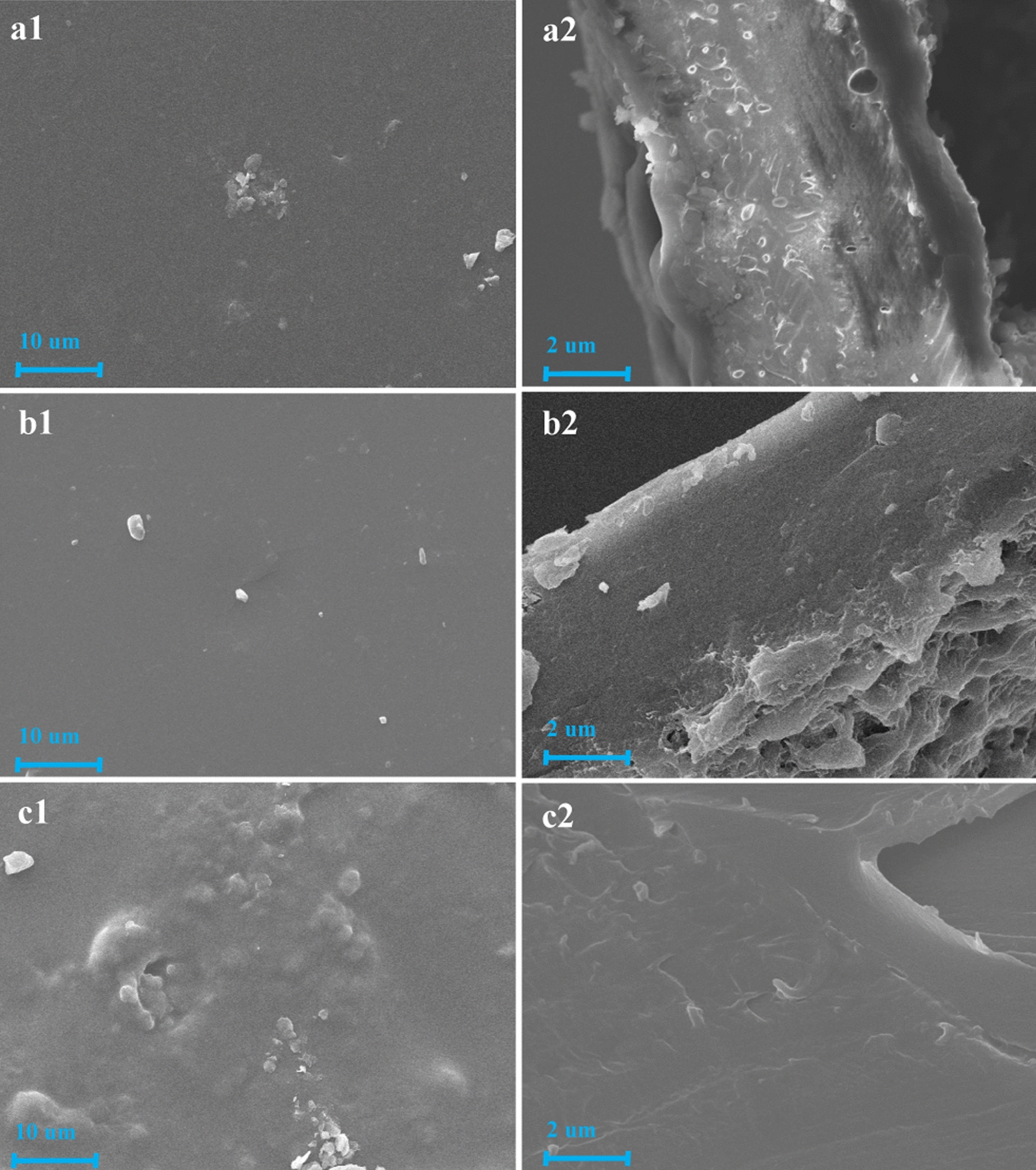
SEM images of the LPUCU (**a1**: 1000×; **a2**: 5000×), EPCU (**b1**: 1000×; **b2**: 5000×), and DLCU (**c1**: 1000×; **c2**: 5000×). Among them, **a1**, **b1**, and **c1** appeared surface images, but **a2**, **b2**, and **c2** showed section images

Different technologies significantly alter the morphology of coating material, which also reflects the property of the coated fertilizer. To investigate the morphology changes caused by the various technologies, the surface (*a*1, *b*1, and *c*1) and section (*a*2, *b*2, and *c*2) images of the LPUCU, EPCU and LDCU were obverted by SEM images (Fig. 5). Most of the surface of the coating of the LPUCU was relatively smooth, and some areas were rough (*a*1). A few holes were observed in the section micrographs of the coating of the LPUCU (*a*2). The holes were easily permeated by water, causing quick release of nutrients from the coated fertilizer. The roughness of the surface of the coating of the LPUCU was due to the fact that the coating materials were inhomogeneously dispersed and agglomerated, because the strong hydrogen bonding in lignin structure. The surface (*b*1) and section (*b*2) of the coating of the EP appeared more smooth, compact, and uniform morphologies than that of the EPCU. When EP was added to the LPU coating material, the surface (*c*1) and section (*c*2) of the coating of the obtained LDCU were more compact and less holes than that of LDCU. In brief, the EP was an effective amendment for the LPU during the preparation of the LDCUs.

**Fig. 6:**
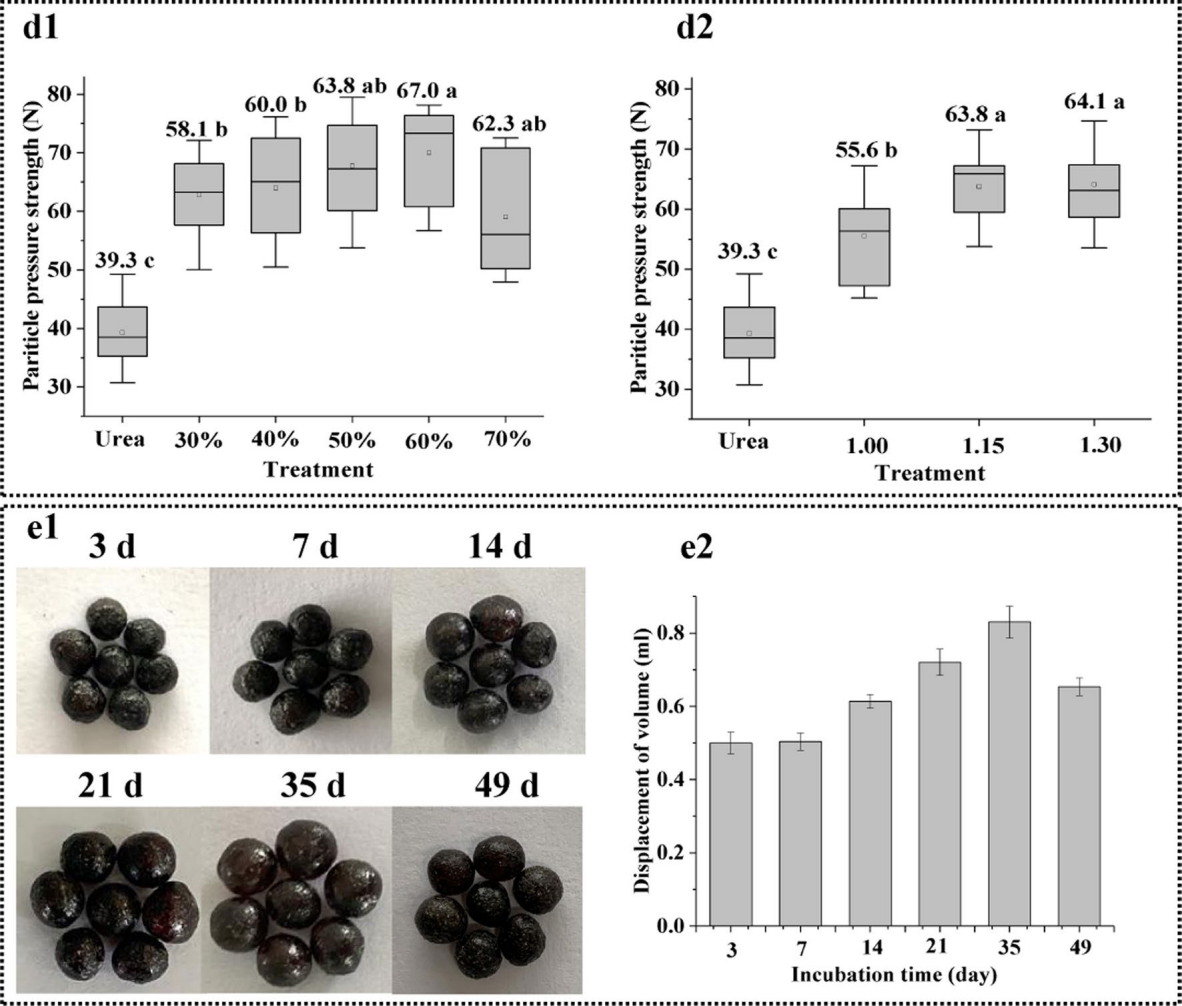
Particle pressure intensity of the DLCU obtained under the different conditions (**d1**: lignin content; **d2**: -CNO/-OH molar ratio). The photo (**e1**) and swelling volume (**e2**) of the LDCU

The particle hardness is critical to the quality of the fertilizer [51]. Compared to the average particle hardness of conventional urea (39.3 N), the average particle hardness of the LDCUs significantly enhanced (55.6–67.0 N) (Fig. 6d1–2). The increasing phenomenon was possibly attributed to the formation of dense coating layer. When the LDCUs were extruded, the particles were not easily broken due to the high hardness [52]. The average particle hardness of the LDCUs first increased from 58.1 N (lignin of 30%) to 67.0 N (lignin of 60%), but then decreased to 62.3 N in the experiment performed at lignin of 70% (Fig. 6d1). There was no significant difference in the average particle hardness of the LDCUs produced using different lignin content. As compared to other LDCUs, the higher average particle hardness (67.0 N) was obtained under lignin of 60% in this study. The results showed that the addition of lignin in the coating layer could improve the compression resistance of particles due to enhanced proportion of hard segments with high-quality aromatic structure [46]. In addition, the –CNO/–OH molar ratio also greatly affected the average particle hardness of the LDCUs (Fig. 6d2). The average particle hardness of the LDCUs gradually increased as the –CNO/–OH molar ratio rose and reached a maximum value (64.1 N). The increased trend suggested that the crosslink density and hard segments were continuously promoted. It was noted that the there was no significant difference in the average particle hardness of the LDCUs obtained under the –CNO/–OH molar ratio exceeded 1.00 conditions. This could be related to hard links that no longer increased due to insufficient –OH with –CNO reaction.

The photo (*e*1) and swelling volume (*e*2) of the LDCUs under different incubation time are shown in Fig. 6. The image change and swelling volume of the LDCUs within 7 days did not change significantly due to the only a small amount of water penetrated into the LDCUs [53]. However, after 7 days, the LDCU granules began to absorb water to dissolve nutrients, which expanded and increased their swelling volumes due to the elevated osmotic pressure difference inside and outside of the coating. The swelling volume of the LDCUs increased by 1.5 times after 35 days compared to the initial fertilizer. Interestingly, the surface of the LDCUs became smoother as the swelling volumes increased, which was similar to the findings of Liu et al. [54]. However, as the incubation time further prolonged to 49 days, the swelling volume of the LDCUs showed a decreasing trend, because the nitrogen concentration in the coating layer reduced after the osmotic pressure difference decreased [55, 56]. In addition, the dissolved urea molecules were released to the outside through the coating layer, which was another reason for the decreasing the swelling volume of the LDCUs [57, 58].

### Release mechanism of nitrogen in the LDCUs

**Fig. 7:**
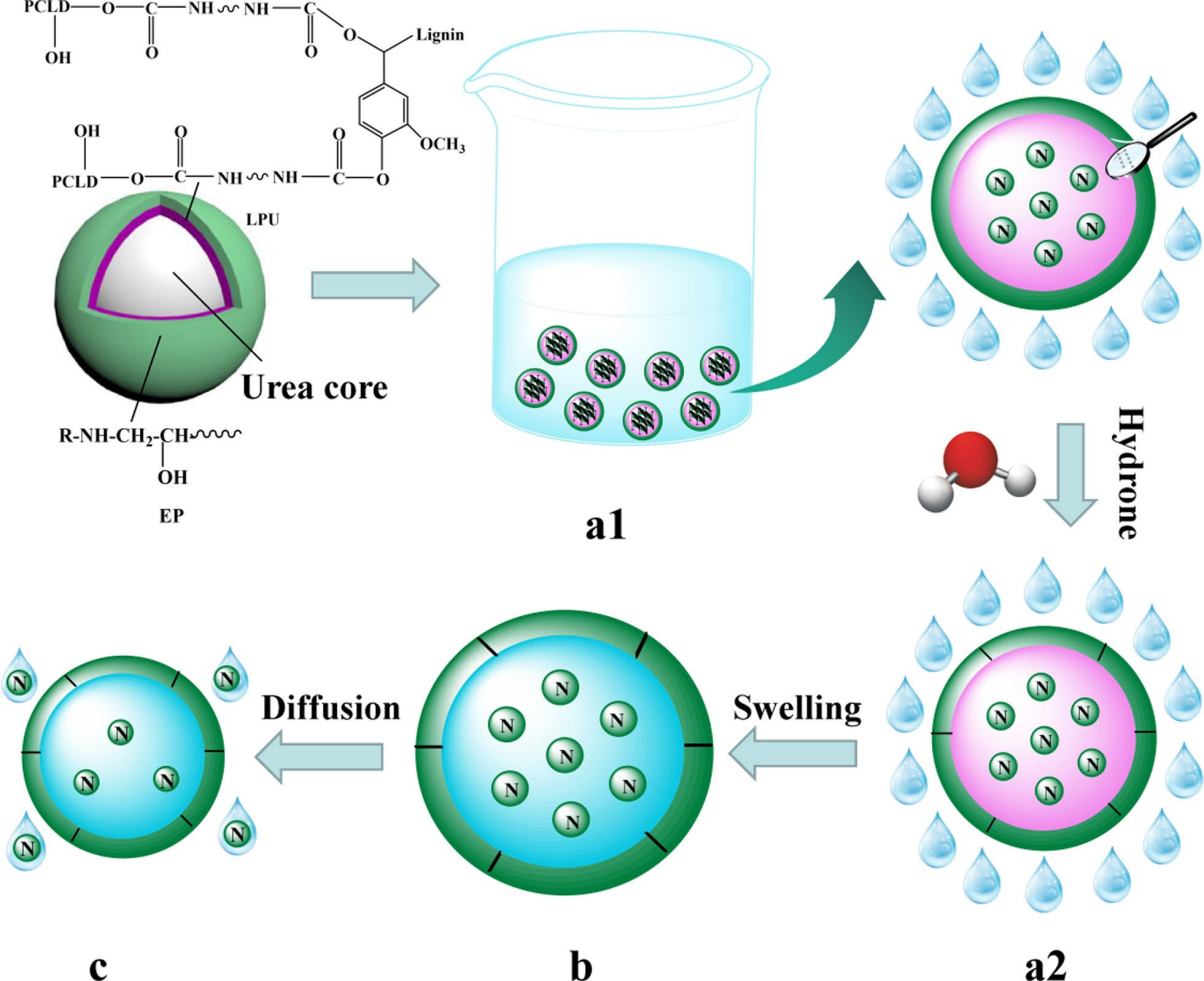
Release mechanism of nitrogen in the LDCU. Initial state of LDCU in water (**a1**); During the initial stage, the hydrone squeezed into the urea core through permeation and diffusion (**a2**); During the urea dissolution stage, the dissolution of urea led to an increase in osmotic pressure and swelling of the coating layer (**b**); During the third stage, the nutrients of urea were released in large quantities through the dissolution process, resulting in a decrease in osmotic pressure (**c**)

Through the above analysis, the release longevity of coated urea was closely related to the preparation parameters of the coating material (lignin content, –CNO/–OH molar ratio, amount of EP, coating rate). For instance, the WCA of the coating material affected nutrient release longevity of the LDCUs [59]. Specifically, the higher WCA of the coating material could result in relatively higher nutrient release longevity of the LDCUs. Likewise, the release longevity of the LDCUs was also positively related to the coating rate [1]. The pores on the surface of the coating increased the water permeability of the coating, which speeded up the dissolution of urea [60]. The nutrient of the LDCUs was released by the penetration and diffusion of hydrone into the inside of the urea particles. The aggregates of hydrone on the fertilizer particles caused the nutrients to dissolve and create an osmotic pressure. The release processes of the LDCUs were mainly divided into three stages (Fig. 7) [61, 62]. During the initial stage, the hydrone squeezed into the urea core through permeation and diffusion (lag period, Fig. 7a2). During the urea dissolution stage, the dissolution rate of urea was significantly faster than the diffusion rate of the urea solution through the coating. The dissolution of urea led to an increase in osmotic pressure and swelling of the coating layer (stable period, Fig. 7b). During the third stage, the nutrients of urea were released in large quantities through the dissolution process, resulting in a decrease in osmotic pressure (recession, Fig. 7c). In addition, the swelling phenomenon of the LDCUs could reflect the release process of nitrogen in the LDCUs to a certain extent. In the early stage of swelling, the volume of the LDCUs did not change. During the middle stage of swelling, the nutrients were dissolved and swelled in the coated urea. As the swelling progresses, the nutrients were expelled from the coating layer, eventually resulting in a continuous decrease in volume of the LDCUs. The holes of the coating layer were easily permeated by water, causing quick release of nutrients from the coated fertilizer. The average particle hardness significantly affected the release performance of the LDCUs. Specifically, when the fertilizer particles were impacted and squeezed, the integrity of the coating layer directly affected the release longevity of nitrogen in the LDCUs [55]. In short, although the nutrient release of the LDCUs was affected by many factors, the successful development of the LDCUs will help improve the rapid development of the coated fertilizer industry.

## Conclusions

A novel green double layer coating with LPU as the inner coating and EP as the outer coating was successfully prepared for coated urea. It was found that the weight loss and WCA (75.6–63.6°) of the LPU simultaneously reduced with the increasing lignin content. The average particle hardness of the LDCUs first increased from 58.1 N (lignin of 30%) to 67.0 N (lignin of 60%), but then decreased to 62.3 N in the experiment performed at lignin of 70%. In addition, the WCA (64.7–75.1°) of the LPU and particle hardness (55.6–64.1 N) of LDCU increased as the –CNO/–OH molar ratio increased. The surface properties and physical properties of the LDCUs were effectively improved. The coating layer of the LDCUs appeared excellent biodegradability in the soil. The optimal cumulative nutrient release rate (79.4%) of LDCU was obtained (lignin of 50%, –CNO/–OH molar ratios of 1.15, EP of 35%, and coating ratio of 5%). The aggregates of hydrone on the LDCU caused the dissolution and swelling of nutrients, and then the diffusion of nutrients through the concentration gradient. The successful preparation of the LDCUs will contribute to the rapid green promotion of the fertilizer industry and the upgrading of products.

## Materials and methods

### Materials

Alkali lignin (AL, solid content of 96.05%, lignin of 93.16%, ash of 3.41%, other substances of 3.43%, sulfur content of 0) with high hydroxyl contents (4.2 mmol/g) was provided by Shandong Xinglong Paper (Group) Co., Ltd. Granular urea (GU, 3–5 mm in diameter and 46.4% N) was purchased from China National Offshore Petrochemical Co., Ltd. Anhydrous pyridine, Deuterated chloroform (99.8%), Hexamethylene diisocyanate (HDI, 99%, wt%), tin 2-ethylhexanoate (95%, wt%) and triethylene tetramine (65%, wt%) were analytical grade and purchased from the Shanghai Macklin Biochemical Co., Ltd. Poly(ε-capro-lactone) diol (PCLD, average *M*_*n*_ ~ 530, hydroxyl value 200 mg KOH/g) was supplied from Shanghai Xushuo Biological Technology Co., Ltd. Epoxy resin-44 (E-44) was also purchased from Nantong Xingchen Synthetic Material Co., Ltd.

### Optimizing experimental design using the surface response model (SRM)

Based on the SRM, the central composite design was implemented using Design-Expert V8.0.6 software. In the response surface method, multiple quadratic regression equations are usually used to fit the functional relationship between factors and response values. It is a common statistical method to solve multivariate problems and find the optimal process parameters through the analysis of regression equations. In this study, the lignin content (*A*), –CNO/–OH molar ratio (*B*), amount of EP (*C*), and coating ratio (*D*) were used as coded variable levels. In addition, − 2, − 1, 0, 1 and 2, respectively, represented the low, medium low, medium, medium high and high levels of the independent variable. The 35 days nitrogen cumulative release rate of LDCU was set as the response values. Table 1 shows the experimental factors and levels. Table 2 lists the 30 experimental designs.

**Table 1:** The experimental factors and levels

Variable	Code	Range and levels				
		− 2	− 1	0	1	2
Lignin content (%)	*A*	10	30	50	70	90
–CNO/–OH molar ratio	*B*	0.85	1.00	1.15	1.30	1.45
Amount of EP (%)	*C*	5	20	35	50	65
Coating ratio (%)	*D*	1	3	5	7	9

**Table 2:** The 30 experimental designs

No	Lignin content *A* (%)	–CNO/–OH molar ratio *B*	Amount of EP *C* (%)	Coating ratio *D *(%)
1	50	1.15	5	5
2	50	1.15	35	5
3	30	1.00	50	7
4	50	1.15	35	5
5	50	1.15	35	9
6	50	1.15	65	5
7	30	1.30	20	7
8	70	1.30	20	3
9	50	1.15	35	1
10	90	1.15	35	5
11	30	1.00	20	3
12	70	1.30	50	7
13	70	1.00	20	3
14	50	1.15	35	5
15	70	1.00	50	3
16	70	1.00	20	7
17	30	1.30	50	7
18	10	1.15	35	5
19	30	1.30	50	3
20	70	1.30	50	3
21	70	1.30	20	7
22	30	1.00	20	7
23	30	1.00	50	3
24	70	1.00	50	7
25	50	1.15	35	5
26	50	1.15	35	5
27	30	1.30	20	3
28	50	1.45	35	5
29	50	1.15	35	5
30	50	0.85	35	5

### Preparation of the lignin-based double-layer coated urea

**Fig. 8:**
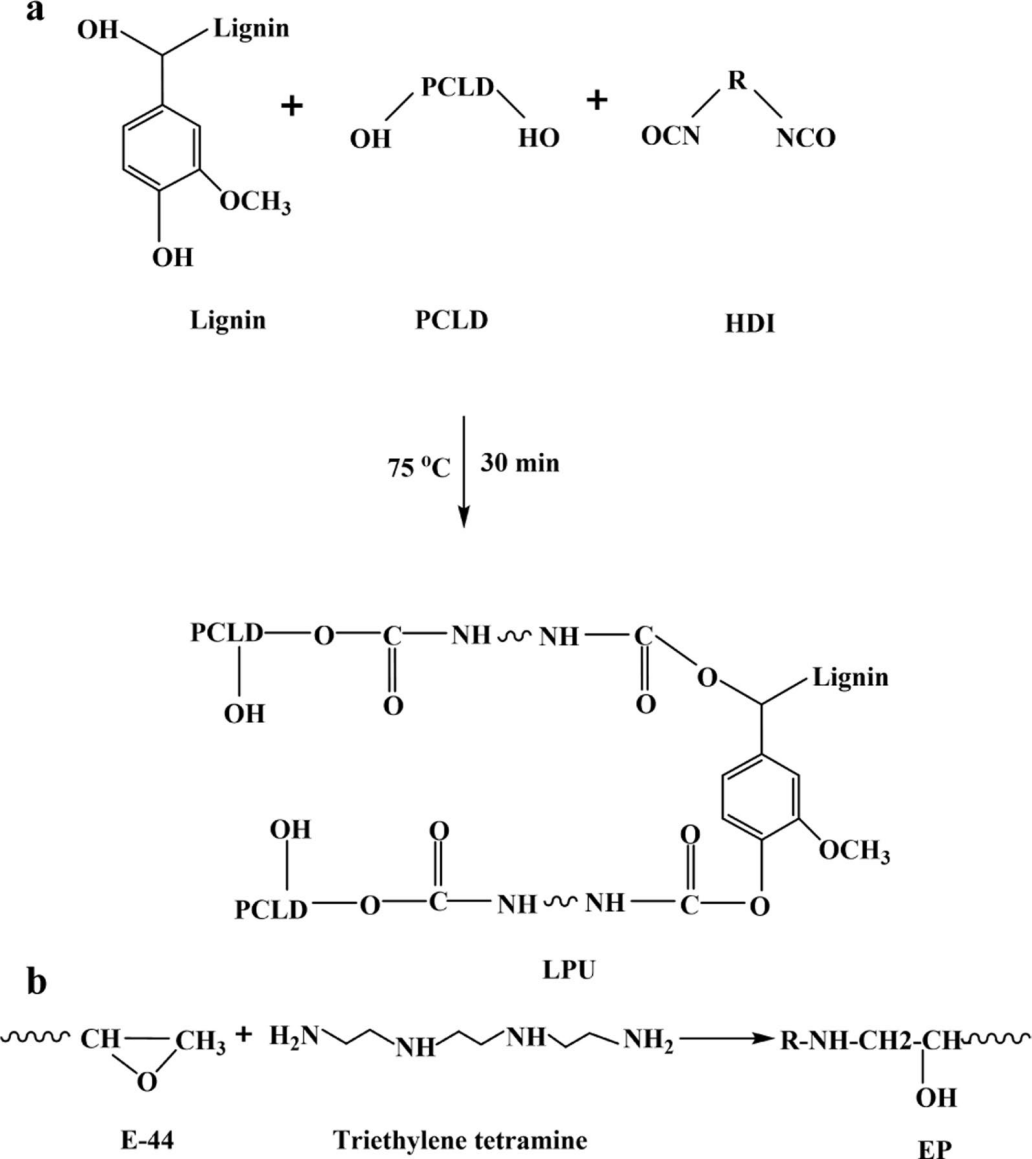
Reaction process of the LPU (**a**) and EP (**b**)

The GU (200 g) was loaded into a rotating drum and then preheated at 75 ± 2 °C for 10 min. After preheating, corresponding to the molar ratio of –CNO/–OH in Table 1, AL was dissolved in PCLD to obtain a bio-based mixed polyol (BMP). The mixed coating composed of BMP and HDI (65% of the total coating materials) were sprayed onto the surfaces of the rotating GU with 300 rpm at room temperature for 5 min. After spraying the mixed coating evenly on the surface of the GU, it was then cured at 75 °C for 30 min to form a single-layer LPU (inner, LPU coating materials) coated urea (Fig. 8a). Subsequently, the same method was used to coat the granules with EP. Specifically, E-44 was softened at 80 °C for 15 min in a water bath. The softened E-44 and triethylene tetramine (accounting for 35% of the total coating materials) with a mass ratio of 4:1 (Fig. 8b) were sprayed onto the surfaces of the rotating LPU coated urea and kept for 10 min (outer, LPU EP coated urea, LECU coating). During the process of LDCU formation, the coating material about 1% of the urea weight (coating rate of 1%) was added to the rotating coated urea. The above process was repeated several times until the coating was complete. Finally, 30 batches of LDCUs under different lignin content, –CNO/–OH molar ratio, amount of EP, and coating ratio were obtained.

### Characterization of the coating materials

To analyze the hydroxyl content of AL, ^31^P NMR spectra of AL was performed as previously reported. 20 mg ALs were dissolved in 0.5 mL of anhydrous pyridine and deuterated chloroform (1.6:1, v/v) under stirring. This was followed by the addition of 0.1 mL cyclohexanol (10.85 mg/mL in anhydrous pyridine and deuterated chloroform 1.6:1, v/v) as an internal standard, and 0.1 ml chromium acetylacetonate solution (5 mg/mL in anhydrous pyridine and deuterated chloroform 1.6:1, v/v) as relaxation reagent. Finally, the mixture were reacted with 0.1 mL phosphorylating reagent 2-chloro-4,4,5,5-tetramethyl-1,3,2-dioxaphospholate was transferred into a 5 mm NMR tube for analysis. Fourier Transform infrared Spectroscopy (FT-IR) spectra of the AL, PCLD, HDI, E44, EP and LPUs were recorded on a Bruker spectrophotometer (VERTEX 70, Germany) with a resolution of 4 cm^−1^. An average of 16 scans ranging from 400 to 4000 cm^−1^ was used. A KBr disk containing 1% finely ground AL was used for determination.

The ^13^C NMR spectra of liquid (AL, PCLD and HDI) were registered on a Bruker Avance 500 spectrometer, equipped with a BBO probe with gradient in *Z* axis. A decoupled sequence zgdc from Bruker library was used at 125.77 MHz. A time domain of 64 k, and a spectral width of 31,000 Hz were used. The interpulse delay was set to 2 s and the acquisition time to 1.5 s. For each spectrum 32,000 scans were accumulated. In addition, LPU was qualitatively characterized by solid state ^13^C-cross-polarization magic angle spinning NMR. The spectra were recorded on a Bruker 400 AVANCE III WB spectrometer 9.40T, using a 4 mm DVT–MAS probe at a spinning rate of 10 kHz. The standard cross-polarization pulse sequence (100.6 MHz), a time domain of 2 k, a spectral width of 29 kHz, a contact time of 1.5 ms and an interpulse delay of 5 s were used.

Thermogravimetric (TGA) analysis of the LPUs was conducted using a thermal gravimetric analyzer (TGA 550, Delaware, USA). The LPUs were heated from 25 to 700 °C at a heating rate of 10 °C/min under highly purified nitrogen atmosphere. Water contact angle (WCA) of the LPUs were performed by sessile-drop method using the contact angle goniometer (JC2000C1, Biolin Scientific, Finland) under room temperature and statistical analysis were repeated three times for averaging. The morphologies of the LPU coated urea (LPUCU), EP coated urea (EPCU) and LDCU were examined using scanning electron microscopy (SEM) (EVO10, Carl Zeiss, Germany). To examine the sections of the LDCU, the samples were cut into two halves and the sections were coated with a gold layer prior to SEM analysis.

### Characterization of the LDCUs under various coating conditions

The nutrient release performance of the LDCUs was determined in water at room temperature. Briefly, a 10.0 g LDCUs was placed into a 250 mL plastic bottle containing 200 mL of distilled water at room temperature. The nitrogen release rate of the LDCUs was recorded after 1, 3, 5, 7, 14, 28, 35, and 42 days by measuring the nitrogen concentration according to the Kjeldahl method [32]. The cumulative nutrient release of the LDCUs after 24 h immersion was defined as the initial release percentage. The experiments were performed in triplicates. The expansion rate of the LDCUs was measured for both before and post-releasing using the drainage method. A 10.0 g LDCU was added into a 10 mL graduated cylinder equipped with 5 mL water to measure their volume (*V*_1_). The LDCU was then subject to the release treatment for 3, 7, 14, 21, 28, 35 or 49 days. The post-release LDCU was measured to determine their volume (*V*_2_). Expansion rate of the LDCU was calculated as: (*V*_2_ − *V*_1_)/*V*_1_ × 100% [33]. The pressure strengths of the LDCUs were analyzed by a particle pressure strength meter (FT-803, Ruike Weiye Instrument Co., Ltd, Zhejiang, China). The coating materials (LPU + EP) of the LDCUs were weighed and placed in a nylon mesh bag (100 mesh, 10 cm × 3 cm) followed by a burial treatment in the soil layer 10 cm deep. The relative humidity was controlled at about 65%, and the test time lasted for 180 days. Among them, the coating layers in the LDCUs were taken every 2 months, washed with tap water to remove soil residues, and then placed in an oven at 80 °C to completely remove water. After the soil burial test, the biodegradation rate of the coating layers in the LDCUs indicated the biodegradability of the sample. The biodegradation rate was calculated by the following equation: *N* = (*M*_0_−*M*_*t*_)/*M*_*0*_ × 100%, where *M*_0_ is the weight of the LDCUs at the initial stage and *M*_*t*_ is the weight of the LDCUs on day.

### Statistical analysis

All statistical analyses were conducted using Excel 2010 and the SPSS Statistical 17.0. Comparisons of various treatments were evaluated using analysis of variance (ANOVA). The differences among means and correlation coefficients were considered significant when *p* < 0.05.

## Abbreviations

TL: Technical lignin
PLA: Polylactic acid
LPU: Lignin-based polyurethane
CRU: Controlled release ureas
EP: Epoxy resin
AL: Alkali lignin
LDCU: Lignin-based double-layer coated urea
GU: Granular urea
HDI: Hexamethylene diisocyanate
PCLD: Poly(*ε*-capro-lactone) diol
SRM: Surface response model
BMP: Bio-based mixed polyol
LECU: Lignin-based polyurethane epoxy resin coated urea
FT-IR: Fourier transform infrared spectroscopy
NMR: Nuclear magnetic resonance
TGA: Thermogravimetric
WCA: Water contact angle
LPUCU: Lignin-based polyurethane coated urea
EPCU: Epoxy resin coated urea
SEM: Scanning electron microscopy
